# Nuclear magnetic resonance-based serum metabolomic analysis reveals different disease evolution profiles between septic shock survivors and non-survivors

**DOI:** 10.1186/s13054-019-2456-z

**Published:** 2019-05-14

**Authors:** Zhicheng Liu, Mohamed N. Triba, Roland Amathieu, Xiangping Lin, Nadia Bouchemal, Edith Hantz, Laurence Le Moyec, Philippe Savarin

**Affiliations:** 1 School of Pharmacy, Anhui Medical University, Hefei, China; 2 Sorbonne Paris Cité, Laboratoire de Chimie, Structures et Propriétés de Biomateriaux et d’Agents Therapeutiques, UMR 7244, University Paris 13, F-93017 Bobigny, France; 3 Intensive Care Unit, Diaconesse-Croix Saint-Simon Hospital, 125 rue d’Avron, 75020 Paris, France; 4 Université Paris Saclay, University Evry, UBIAE EA 7362, 91025 Evry, France

**Keywords:** ^1^H nuclear magnetic resonance spectroscopy, Metabolomics, Septic shock, Outcome prediction

## Abstract

**Background:**

Septic shock is the most severe phase of sepsis and is associated with high rates of mortality. However, early stage prediction of septic shock outcomes remains difficult. Metabolomic techniques have emerged as a promising tool for improving prognosis.

**Methods:**

Orthogonal projections to latent structures-discriminant analysis (OPLS-DA) models separating the serum metabolomes of survivors from those of non-survivors were established with samples obtained at the intensive care unit (ICU) admission (H0) and 24 h later (H24). For 51 patients with available H0 and H24 samples, multi-level modeling was performed to provide insight into different metabolic evolutions that occurred between H0 and H24 in the surviving and non-surviving patients. Relative quantification and receiver operational characteristic curves (ROC) were applied to estimate the predictability of key discriminatory metabolites for septic shock mortality.

**Results:**

Metabolites that were involved in energy supply and protein breakdown were primarily responsible for differentiating survivors from non-survivors. This was not only seen in the H0 and H24 discriminatory models, but also in the H0-H24 paired models. Reanalysis of extra H0-H24 paired samples in the established multi-level model demonstrated good performance of the model for the classification of samplings. According to the ROC results, nine discriminatory metabolites defined consistently from the unpaired model and the H0-H24 time-trend change (Δ_H24-H0_) show good prediction of mortality. These results suggest that NMR-based metabolomic analysis is useful for a better overall assessment of septic shock patients.

**Conclusions:**

Dysregulation of the metabolites identified by this study is associated with poor outcomes for septic shock. Evaluation of these compounds during the first 24 h after ICU admission in the septic shock patient may be helpful for estimating the severity of cases and for predicting outcomes.

**Trial registration:**

All human serum samples were collected and stored, provided by the “center of biologic resources for liver disease”, in Jean Verdier Hospital, Bondy, France (BB-0033-00027).

**Electronic supplementary material:**

The online version of this article (10.1186/s13054-019-2456-z) contains supplementary material, which is available to authorized users.

## Background

Septic shock is the most severe phase of sepsis [1, 2]. It is defined as sepsis complicated either by hypotension that is refractory to fluid resuscitation or by hyperlactacidemia and is often accompanied by acute organ failure. Mortality rates associated with septic shock are 20 to 30% in many series, principally due to multiple organ dysfunction syndrome (MODS) [3]. Common strategies for the treatment of septic shock include prompt initiation of therapy to treat the underlying infection with antibiotics, vasopressor therapy, and support for failing organs. In recent years, early goal-directed therapy (EGDT), which improves curative effect, has been extensively applied to improve rescue outcomes [4, 5]. However, early personalized prognosis and diagnosis remain challenging due to the complicated etiology and pathogenesis of septic shock. Determination of an acute prognosis in the early stage of sepsis is of great importance to improve therapeutic efficacy and will aid in the development of adapted strategies for different cases. In fact, evaluation of existing biomarkers (e.g., TNF-α, IL-6, and PCT) and clinical scores such as the sequential organ failure assessment (SOFA) [6] have been applied prognostically but their performance (sensitivity, specificity) has not proven adequate for all cases [7]. Thus, new methods for reliable early prognosis are still urgently needed.

Metabolomics has been proven to be a promising tool that aid in the prognosis of sepsis. This is because metabolomics allows to provide comprehensive information of personalized metabolome and therefore to enable the prediction of personalized outcome for septic patients. Previous studies have shown that there are considerable differences in the metabolome fingerprints between septic shock survivors and non-survivors. However, notably, most of the previous studies in human septic patients were designed to be performed by analysis of one unique sampling, and no studies have derived dynamic alterations of patient metabolomes during clinical therapy. However, good outcomes for septic shock are associated with a less severe disease course and a positive therapeutic response to treatment. In a previous study, we reported comprehensive differences in the metabolic profiles between septic shock survivors and non-survivors at the admission to the intensive care unit (ICU), based on a liquid chromatography-mass spectrometry (LC-MS) approach [8]. In this current study, samples from the septic shock patients which were obtained 24 h after ICU admission were also included. The aim of the present study was to analyze the discriminatory ability of metabolic profiles between septic shock survivors and non-survivors at the beginning and 24 h after ICU admission and also to describe the evolution of metabolic profiles for septic shock patients during this period by using ^1^H NMR spectroscopy-based metabolomics.

## Materials and methods

### Patient inclusion

Between January 2009 and December 2011, all consecutive adults admitted to our intensive care unit were enrolled in this study if they had an indisputable or probable septic shock in the first 24 h after ICU admission [9]. Septic shock was defined as the presence of a clinically or microbiologically documented infection and on-going treatment with vasopressor therapy (norepinephrine or epinephrine at a dose ≥ 0.25 μg per kilogram of body weight per minute or at least equal to 1 mg per hour) for at least 6 h to maintain a systolic blood pressure of at least 90 mmHg or a mean blood pressure of at least 65 mmHg. Non-inclusion criteria were (i) patient younger than 18 years, (ii) patient with solid cancer or blood cancer, and (iii) patient with liver cirrhosis or chronic kidney disease. Patients were treated according to the international guidelines for the management of sepsis and shock septic [5].

Biological parameters, hemodynamic parameters, and the use of catecholamine and mechanical ventilation were recorded at inclusion. Cause of septic shock was recorded. To evaluate the severity of the disease, the Sequential Organ Failure Assessment (SOFA) score was calculated during the first day of admission [10]. ICU and hospital length of stay and mortality were recorded. The survival status of each patient was noted 7 days after the first sample.

### Sample collection

All the first samplings (H0) were obtained withdrawn just before or immediately after clinical vasopressor therapy initiation on the patients. The second samples were withdrawn 24 h after the beginning of the vasopressor introduction. Blood samples were collected in serum separator tubes (SST). SST were stored for at least 30 min and not more than 1 h and 30 min. After centrifugation (1000×*g*, 25 °C, 10 min), the serum was stored at −80 °C. All human serum samples were collected and stored, provided by the “center of biologic resources for liver disease”, in Jean Verdier Hospital, Bondy, France (BB-0033-00027). Written informed consent was obtained from all subjects or their surrogate decision-maker. The local ethics committee approved the protocol.

### Regrouping and matching of samples

**Fig. 1:**
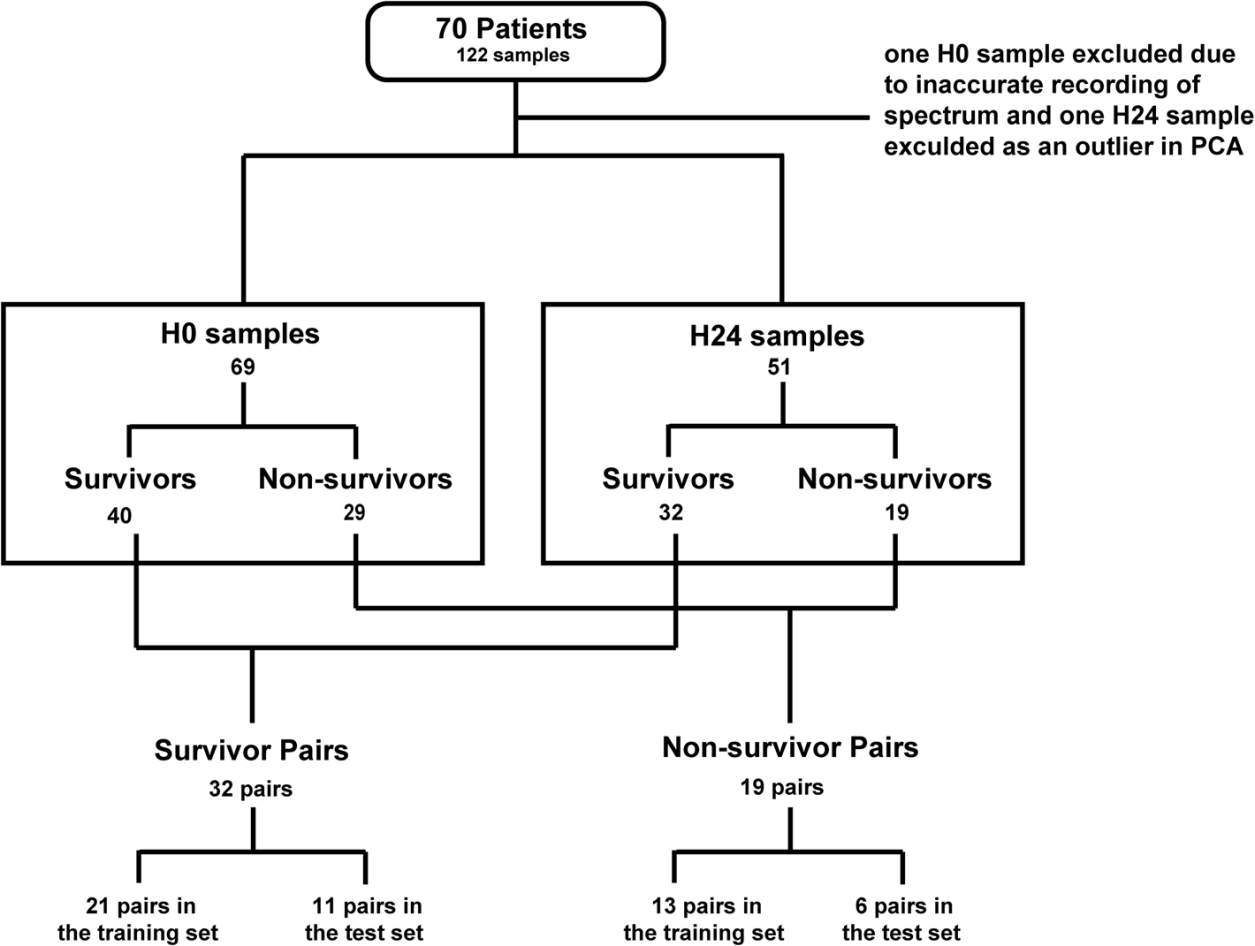
Regrouping and matching of samples. One H24 sample from a survivor was excluded due to the problem of NMR gain parameter; one H0 sample from a non-survivor was excluded as it was found to be an outlier in PCA. For the paired H0-H24 samples obtained from the same patients, the pairs have been divided into training set and test set. The pairs of survivors and non-survivors were analyzed separately. Samples in the training set were analyzed for establishing discriminatory models between H0 and H24 samples. The pairs in the test set were reanalyzed in the established models

As shown in Fig. 1, 122 samples from 70 patients were obtained. Seventy samples were drawn at ICU admission and are noted as H0 samples. 52 samples were obtained 24 h after the first sampling and are noted as H24 samples. During analysis, one H0 sample from a non-survivor who did not have a matching H24 sample was excluded as a spectral outlier. One H24 sample from a survivor was excluded while the H0 sample that belonged to the same patient was retained. The exclusion of the sample was due to the drastically affected NMR gain parameter. The spectrum of this sample was therefore found to be clearly different from the others. Among the non-survivors, 11 patients died prior to the H24 sampling and their H24 samples were therefore not available. For the other samples, each H24 sample was matched with the H0 sample which was collected from the same patient. In this case, 32 pairs for survivors and 19 pairs for non-survivors were obtained. For both septic shock survivor (SSS) and non-survivor (SSN) H0-H24 pairs, two thirds were randomly taken into the training set while the remaining were put into the test set.

### Sample preparation and NMR data acquisition

Samples were defrosted at room temperature. A volume of 450 μL of each sample was diluted with 50 μL of D_2_O in an NMR tube of 5 mm diameter. All the samples were then analyzed with a 500-MHz NMR spectrometer (Advance III, Bruker, Germany) at 297 K. The free induction decay (FID) signals were collected onto 64k data points, with a spectral width of 6000 Hz. The 1D ^1^H NMR spectra were recorded by the Carr-Purcell-Meiboom-Gill (CPMG) sequence [11] with 128 transients for each spectrum. For several samples, 2D NMR experiments (TOCSY and JRES sequences) were achieved to confirm spectral assignments. The mixing time of the TOCSY spectra was 80 ms with 32 transients.

### Data processing

After the FIDS were acquired for all the samples, they were processed using the NMRPipe software [12]. All FIDs were multiplied by a 0.3-Hz exponential line broadening factor prior to Fourier transformation. Phasing of each spectrum was manually adjusted, and baselines were corrected using a linear method. All the spectra were divided into 0.001 ppm buckets between − 1 and 10 ppm. The residual water signal (4.6 to 5.5 ppm) was excluded, and the spectral region from 3.16 to 4 ppm was also removed since signals observed in this section represented the infusion of hydroxyethyl starch (HES), which was applied in the ICU to heighten blood tension for the patients who suffered from hypotension. The spectra were then normalized using the probabilistic quotient method [13]. All the buckets were centered by the method of auto-scaling. The peaks were adequately assigned using the Human Metabolome Database (HMDB, www.hmdb.ca) NMR library, the Chenomx software (Chenomx Inc., Canada), and the 2D experiments. As to the annotation of NMR peaks, an exemplar NMR spectrum has been shown in Additional file 1: Figure S1 with some of the assignments.

### Statistical analyses

All the multivariate analyses were achieved using an in-house code which is based on the code of Trygg and Wold [14], developed using Matlab software (version 2012b, MA, USA). Prior to the establishment of discriminatory analyses, a principal component analysis (PCA) with H0 samples from all the included non-survivors shows that the main variability among these samples does not correspond to the time of the death, as shown in Additional file 1: Figure S2. Another PCA for all the acquired spectra was performed to ensure that there were no outliers (Additional file 1: Figure S3). Orthogonal projections to latent structures-discriminant analyses (OPLS-DA) were performed for differentiating survivors from non-survivors with H0 and H24 samples, respectively. Samples obtained at H0 and H24 from the same patients were paired and analyzed in multilevel models to study the interindividual variability [15]. The paired samples were divided into survivor and non-survivor groups. Two OPLS-DA multilevel models were applied with the survivors and non-survivors, respectively, separating H0 from H24 samples. The models were all validated by cross-validation with 500 permutations of variable *X* and *Y*, where *X* represents the data matrix and *Y* represents the discriminatory variable for each model [15, 16]. For the univariate analyses analyzing the significant differences between two groups, the *P* values were calculated with Student’s *T* test. The false discovery rate (FDR) was calculated by the Multiple Experiment Viewer Toll (version 4.9.0, OriginLab, Northampton, USA); the correction of *P* value is performed with “adjusted Bonferroni correction” [17]. The threshold of FDR was set at 0.1 for the screening of the discriminatory metabolites, that is, the variables with FDRs superior to 0.1 were not considered as important discriminants. A significant difference between compared groups was defined with an adjusted *P* value inferior to 0.05.

## Results

### Baseline characteristics of patients

The baseline biological characteristics of all the included patients are shown in Table 1. Partial pressure of arterial oxygen (PaO_2_) and the ratio of PaO_2_ to the percentage of inspired oxygen (FiO_2_) were significantly different between the survivors and non-survivors at H0. Lactate level in non-survivors was also found to be significantly increased than those in survivors. For the clinical scores, SAPSII and SOFA, SAPSII was able to discriminate between survivors and non-survivors. However, due to the sample size, it was not satisfying to predict mortality with SOFA in this study, according to the results both at H0 and H24.

**Table 1:** Baseline characteristics of the patients recorded at admission to the ICU

	Total/average	Survivors	Non-survivors	Adj *P*	FDR
Number of patients	70	40	30		
Male (%)	40 (57%)	27 (67%)	13 (32%)	0.07	0.09
Age	70.1 ± 0.16	68.5 ± 0.29	72.1 ± 0.36	0.12	0.23
Temperature (°C)	37.3 ± 0.02	37.1 ± 0.03	36.9 ± 0.05	0.32	0.35
Mean arterial pressure (mmHg) at admission	72.8 ± 2,44	71,0 ± 3,25	75.2 ± 3.72	0.41	0.35
pH at admission	7.3 ± 0.00	7.31 ± 0.00	7.29 ± 0.00	0.43	0.53
Pa_O2_ (mmHg)_H0_	144.7 ± 1.54	167.5 ± 2.83	113.6 ± 3.12	0.05	0.13
Pa_CO2_ (mmHg)	37.6 ± 0.18	37.6 ± 0.31	37.5 ± 0.45	0.66	0.63
Pa_O2_/FiO_2_ ratio_H0_	212.5 ± 4.10	168.6 ± 3.19	242.2 ± 5.59	0.04	0.07
Lactate (mmol/L)_H0_	5.0 ± 0.07	3.7 ± 0.11	6.6 ± 0.17	0.01	0.03
Creatininemia (μmol/L)_H0_	212.2 ± 3.88	200.1 ± 4.34	241.9 ± 3.55	0.07	0.15
Glycemia (mmol/L)	9.8 ± 0.09	9.9 ± 0.20	9.6 ± 0.23	0.91	0.95
Hemoglobin (mmol/L)	10.5 ± 0.03	10.1 ± 0.06	10.9 ± 0.07	0.09	0.06
Albumin (g/L)	24.6 ± 0.13	22.2 ± 0.15	26.8 ± 0.38	0.11	0.21
Platelet (g/L)	156.7 ± 1.53	151.8 ± 2.61	162.2 ± 3.70	0.33	0.13
Total bilirubin (μmol/L)	38.4 ± 0.90	37.8 ± 1.90	39.1 ± 1.53	0.54	0.77
CRP (mg/dL)	162.3 ± 2.15	174.0 ± 3.98	147.4 ± 4.71	0.49	0.67
PCT (mg/dL)	25.2 ± 0.52	28.0 ± 1.15	21.6 ± 1.39	0.74	0.88
SAPSII	59.0 ± 0.24	55.2 ± 0.39	64.3 ± 0.58	0.02	0.07
SOFA_H0_	11.7 ± 0.06	10.9 ± 0.11	12.4 ± 0.12	0.10	0.15
SOFA_H24_	9.4 ± 0.06	8.7 ± 0.11	10.3 ± 0.10	0.07	0.11
ICU LOS (day)	9.16 ± 1.21	15.1 ± 1.36	3.62 ± 0.09	0.05	0.09
Mechanical ventilation (%)	84%	75%	93%		
Hospital-acquired infection (%)	45%	35%	60%		
Sepsis causes (%)					
Pulmonary	54%	55%	53%		
Abdominal	30%	22%	40%		
Urinary tract	7%	7%	6%		
Others	8%	15%	0%		

All the data is represented as mean ± standard error of mean (SEM)

*PaO2* partial pressure of arterial oxygen, *FiO2* percentage of inspired oxygen, *SOFA* Sepsis-related Organ Failure Assessment, *SOFA*_*H0*_ SOFA measured at H0, *SOFA*_*H24*_ SOFA measured at H24, *SAPSII* new simplified acute physiology score, *LOS* length of stay, *FDR* false discovery rate, *Adj P P* value adjusted with Bonferroni correction

### Discriminatory analyses separating septic survivors from non-survivors with samples drawn before treatment (H0)

**Fig. 2:**
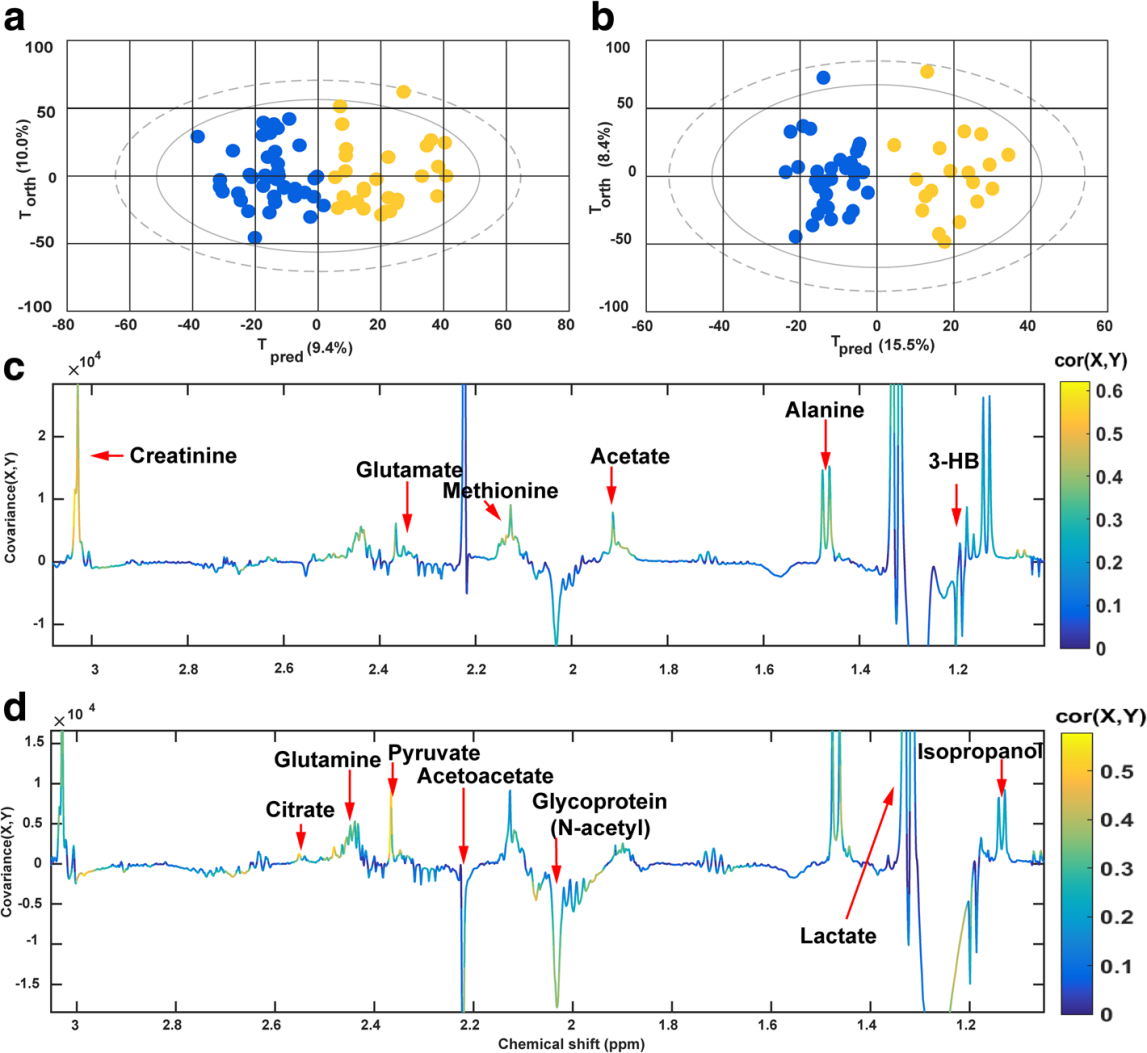
OPLS-DA between septic shock survivors and non-survivors at H0 and H24. **a**, **b** Score plots for the H0 and H24 models, respectively. Blue dots represent the survivors and yellow dots represent the non-survivors. T_pred_: The components that predict the differences between the groups; T_orth_: components that do not predict the differences between the groups; **c**, **d** Loading plot for the H0 and H24 models, respectively. The color of the peaks indicates the correlation between the marked peak and the classification of the sample. Colors that are close to red correspond to a higher correlation. Positive peaks in the loading plot correspond to metabolites which increased in non-survivors; negative peaks correspond to metabolites that decreased in non-survivors

For the H0 samples, a total of 69 samples were analyzed using an OPLS-DA model (PCA models separating the survivors from the non-survivors prior to the exclusion of the outlier have been illustrated in Additional file 1: Figure S3). Among these, 40 samples were obtained from survivors and 29 were from non-survivors. As is shown in Fig. 2a for H0, a clear separation between the two groups of patients is demonstrated by the score plot. The *Q*^2^*Y*, which indicates the predictability of the model, was equal to 0.60 with three components, and the *R*^2^*Y*, which indicates the fraction of explained variance of the *Y* variable, was 0.75, where *Y* corresponded to the survival condition in the model for the SSS vs. SSN comparison. Cross-validation showed that the model was not over-fitted (Additional file 1: Figure S4). In the loading plot (Fig. 2c), the peaks are colored according to the correlation coefficients, which relate to their contribution to the discriminatory model. Corresponding discriminatory metabolites have been listed in Table 2 with their chemical shifts, multiplicity, correlations, variance importance projections (VIPs), and *P* values. The concentrations of various amino acids such as alanine, glutamate, glutamine, methionine, and aromatic amino acids were increased in the non-survivors as compared to the survivors. Significant variations between the two groups were also found in energy-associated metabolites including two tricarboxylic acid (TCA) cycle intermediates, citrate and fumarate, and lactate and pyruvate. Ketone bodies, 3-hydroxybutyrate, and acetate were also elevated in the non-surviving patients. The only decreased signal was observed for the *N*-acetyl moieties of glycoproteins. Together, the results showed considerable differences in the metabolic profiles between the survivors and the non-survivors at H0.

**Table 2:** Metabolites found to discriminate between SSS and SSN at H0

Peaks	Assignment	VIP	Correlation	Adj *P*	FDR
1.06^d^	3-Hydroxyisobutyrate	3.06	0.52	0.0001	0.0001
5.79^s^	Urea	2.64	0.45	0.002	0.003
7.31^m^ 7.36^m^	Phenylalanine	2.64	0.44	0.01	0.01
2.12^m^, 2.32^m^	Glutamate	2.6	0.44	0.02	0.02
2.43^m^	Glutamine	2.55	0.43	0.03	0.01
3.03^s^	Creatinine	2.43	0.41	0.03	0.04
1.32^d^ 4.11^q^	Lactate	2.38	0.4	0.02	0.04
2.14^s^	Methionine	2.17	0.37	0.06	0.05
1.46^d^	Alanine	2.12	0.26	0.07	0.08
6.88^d^ 7.18^d^	Tyrosine	2.02	0.34	0.03	0.04
2.36^s^	Pyruvate	2.01	0.34	0.03	0.01
2.52^d^ 2.62^d^	Citrate	1.94	0.33	0.03	0.04
1.7^m^	Lysine	1.91	0.27	0.09	0.08
6.52^s^	Fumarate	1.9	0.32	0.04	0.05
7.67^s^	1-Methylhistidine	1.66	0.28	0.07	0.06
2.03^s^	Glycoprotein (*N*-acetyl)	1.64	− 0.28	0.08	0.03
1.91^s^	Acetate	1.56	0.25	0.09	0.10
1.16^d^	Isopropanol	1.53	0.26	0.09	0.03

Chemical shifts for the assigned metabolites are shown in the peak column. The superscripts for the peaks represent the multiplicity of the peaks. s, singlet; d, doublet; t, triplet; q, quadruplet; m, multiplet. A positive correlation indicates an increased level of the metabolite in the non-survivor while negative correlation indicates a decreased level of the metabolite. The threshold of FDR was set at 0.1. Similar expressions are also applied for Tables 3 and 4

*Adj P P* values that are calculated by Student’s *T* test are adjusted with Bonferroni correction, *FDR* false discovery rate

### Discriminatory analyses separating septic survivors from non-survivors with samples drawn 24 h after ICU admission (H24)

A second OPLS-DA model differentiating metabolic profiles of SSS from those of SSN were performed with 51 H24 samples. Of these samples, 19 non-survivors were compared with 32 survivors. As shown in Fig. 2b, a separation between SSS and SSN was observed. The *R*^2^*Y* and *Q*^2^*Y* values in the model were equal to 0.86 and 0.46, respectively, and were calculated with three components. The validation by permutations is shown in Additional file 1: Figure S5. Significant discriminant metabolites were identified referring to the loading plot (Fig. 2d) and are listed in Table 3. Interestingly, increasing levels of most amino acids and energy-related metabolites, as well as the decreases of *N*-acetyl moieties of glycoproteins, were still detected in SSN, compared with SSS, in line with the findings of the H0 model. Besides, an increase in ketone bodies and diminishing lipid-related signals were only present at H24, but not at H0, in the non-survivors when compared to survivors. Both H0 and H24 unpaired models revealed extensive variations in the metabolic profiles between SSS and SSN at the admission and 24 h after ICU admission.

**Table 3:** Metabolites found to discriminate between SSS and SSN at 24 h after admission to ICU

Peaks	Assignment	Correlation	VIP	Adj *P*	FDR
2.37^s^	Pyruvate	0.52	3.55	0.0001	0.0001
2.52^d^ 2.62^d^	Citrate	0.52	3.5	0.0002	0.0003
7.31^m^ 7.36^m^	Phenylalanine	0.48	3.25	0.001	0.001
6.88^d^, 7.18^d^	Tyrosine	0.45	3.03	0.004	0.004
2.72^m^	Lipids (fatty acid residues)	− 0.44	2.99	0.01	0.01
2.43^m^	Glutamine	0.44	2.96	0.01	0.01
1.32^d^, 4.41^q^	Lactate	0.44	2.9	0.01	0.02
1.06^d^	2-Hydroxyisovalerate	0.41	2.77	0.02	0.03
3.03^s^	Creatinine	0.37	2.51	0.03	0.05
2.03^s^	Glycoprotein (*N*-acetyl)	− 0.37	2.48	0.05	0.05
7.03^s^ 7.67^s^	1-Methylhistidine	0.35	2.37	0.06	0.07
2.12^m,^2.33^m^	Glutamate	0.33	2.22	0.07	0.09
1.7^m^	Lysine	− 0.29	1.95	0.09	0.07
1.46^d^	Alanine	0.28	1.9	0.13	0.10

### Discriminatory analysis of the evolution of septic shock from H0 to H24 for septic shock survivors and non-survivors

On the basis of the separation found between SSS and SSN within the above two discriminant models, we hypothesized that the therapeutic response between SSS and SSN could be different. To verify this hypothesis, SSS and SSN groups were compartmentalized and studied by two multi-level OPLS-DA models which focused on the intraindividual variability of the metabolome between H0 and H24. The pairwise distance of metabolome variations between H0 and H24 samples were analyzed for the patients for whom both H0 and H24 samples were collected.

**Fig. 3:**
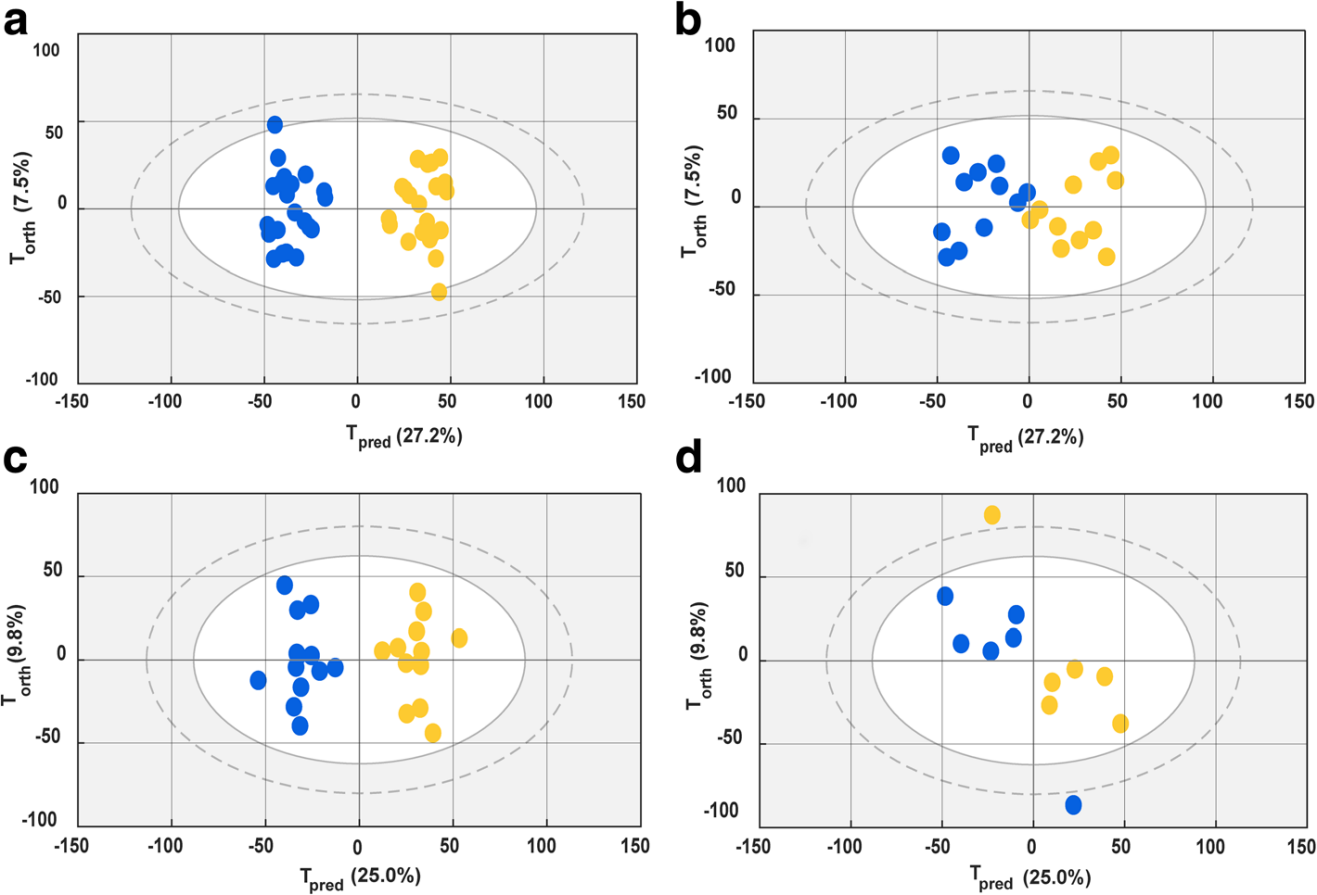
Score plots of OPLS-DA separating H0 from H24. For the patients whose H0 and H24 are both available, their H0 and H24 samples are matched in the discriminatory models. The pairs from the survivors and non-survivors are analyzed in two separated paired models. Blue dots represent the H24 samples and yellow dots represent the H0 samples. **a** Score plot for the training set separating H0 from H24 for the survivors. **b** Reanalysis of test set samples of survivors in the established H0-H24 multi-level model. **c** Score plot for the training set separating H0 from H24 for the non-survivors. **d** Reanalysis of test set samples of non-survivors in the established H0-H24 multi-level model

For the SSS group, 21 pairs were randomly included to establish a model separating the H0 sample from the H24 sample, as shown in Fig. 3a. The *Q*^2^*Y* was 0.78 with 2 components, and *R*^2^*Y* was 0.94. This model was subsequently applied to 11 other pairs. The predictions for these pairs are shown in Fig. 3b. *R*^2^*Y* for the prediction was 0.76, showing a prominent prediction of the classification among H0 and H24 samples. For the SSN model, separation between H0 and H24 samples was also observed, as shown in Fig. 3c. Thirteen pairs were used to set up a training model, and 6 pairs were included in the test set. Consequently, *R*^2^*Y* and *Q*^2^*Y* were 0.57 and 0.91, respectively, and *R*^2^*Y* for the reanalysis was 0.33 (as shown Fig. 3d). The loading plots for the two paired models are shown in Additional file 1: Figure S6. Metabolites which are listed in Table 4 exhibited opposite H0-H24 metabolome evolutions between SSN and SSS. Accordingly, increases of amino acids, energy-related metabolites, and creatinine and a decline of glycoprotein could be observed during the evolution from H0 to H24 for the non-survivors. However, this was not the case for survivors.

**Table 4:** Discriminatory metabolites with different variations along the H0-H24 evolution between the non-survivor group and the survivor group

Peaks	Assignment	C_1_	Adj P_1_	FDR_1_	V_1_	C_2_	Adj P_2_	FDR_2_	V_2_
2.12^m^ 2.32^m^	Glutamate	− 0.62	0.0001	0.001	↓	0.49	0.03	0.02	↑
2.52^d^ 2.66^d^	Citrate	− 0.59	0.0001	0.001	↓	0.59	0.002	0.004	↑
7.32^d^ 7.36^d^	Phenylalanine	− 0.53	0.0004	0.003	↓	0.4	0.08	0.07	↑
2.07^m^ 2.43^m^	Glutamine	− 0.49	0.001	0.01	↓	0.44	0.03	0.03	↑
1.47^d^	Alanine	− 0.42	0.004	0.03	↓	0.42	0.05	0.06	↑
1.32^d^ 4.11^q^	Lactate	− 0.62	0.0001	0.002	↓	0.2	0.26	0.21	NS
2.37^s^	Pyruvate	− 0.38	0.04	0.05	↓	0.06	0.42	0.33	NS
2.03^s^	Glycoprotein (*N*-acetyl)	0.26	0.08	0.09	NS	− 0.48	0.01	0.01	↓
3.02^s^	Creatinine	− 0.21	0.15	0.13	NS	0.47	0.02	0.03	↑

C_1_, correlation of the metabolite to the discriminatory model for the survivors; C_2_, correlation of the metabolite to the discriminatory model for the non-survivors. For each listed metabolite, the sign of C_1_ is opposite to that of C_2_; Adj P_1_, adjusted *P* value (with Bonferroni correction) of the metabolite in the comparison between H0 and H24 samples for the survivors; Adj P_2_, adjusted *P* value of the metabolite in the comparison between H0 and H24 samples for the non-survivors; FDR_1_, false discovery rate for the *P* value calculated with the survivors; FDR_2_, false discovery rate for the *P* value calculated with the non-survivors; V_1_, variation in concentration for the metabolites from H0 to H24 for the survivors; V_2_, variation in concentration for the metabolites from H0 to H24 for the non-survivors; ↑, increased concentration of the metabolite at H24 compared with H0; ↓, decreased concentration of the metabolite at H24 compared with H0. NS, non-significant (Adj *P* > 0.05) variation

### Discrimination between SSS and SSN based on the relative quantification of key discriminators

**Fig. 4:**
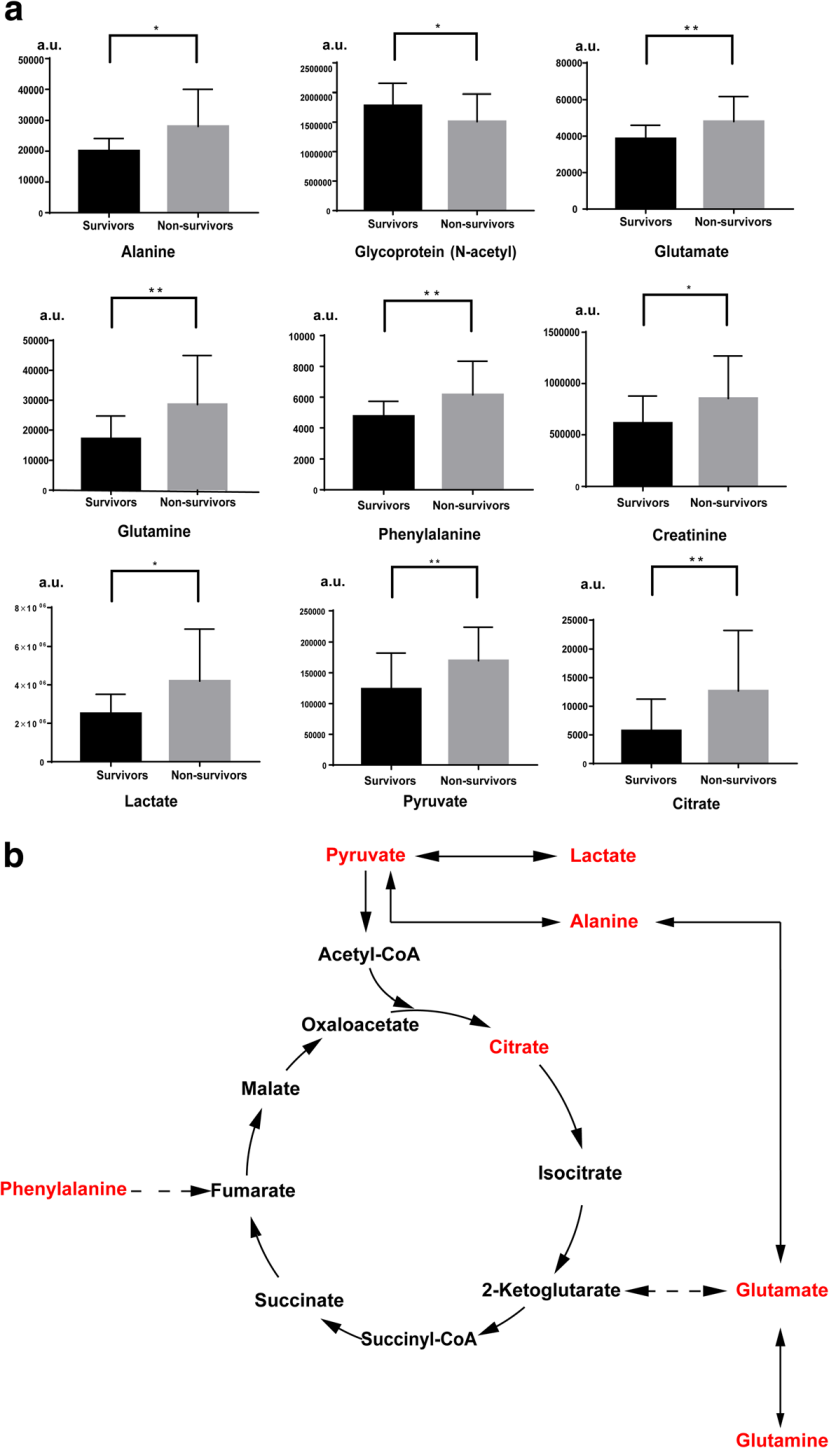
Levels of key discriminatory metabolites and their relevant metabolic pathway in the comparison between SSS and SSN patients during the H0-H24 evolution. **a** Levels of key discriminatory metabolites in the SSS and in SSN. The levels of the metabolites are calculated with the average of time-trend change (Δ_H24-H0_). Averages of Δ_H24-H0_ for the survivors and non-survivors have been respectively shown and calibrated by the standard deviation. *: *p* < 0.05. **: *p* < 0.01. a.u.: arbitrary unit; **b** relevant metabolic pathway for energy-related metabolites and amino acids that vary differentially between SSS and SSN in the H0-H24 evolution. Metabolites marked by red color are those that increase in the SSN compared to SSS in all the models. Solid flashes express a direct conversion between two metabolites and dotted lines represent undirect conversions between two metabolites, according to KEGG metabolic pathway database

Spectral signals corresponding to the metabolites in Table 4 were integrated for the spectra of paired samples. As shown in Table 4, the molecules varied oppositely during the H0-H24 evolution between the survivors and non-survivors. The time-trend change of area, ΔSignal area_H24-H0_, was calculated for each metabolite. Average values for ΔSignal area_H24-H0_ of involved metabolites resulting from the SSN model were compared to those from the SSS model, as shown in Fig. 4a. Interestingly, the metabolites were also shown to be discriminant variables in the comparison between SSS and SSN in previously mentioned H0 and H24 unpaired models. We further calculated the area under the ROC curve for the metabolites in order to test their performance in the classification of surviving patients. ROCs for the discriminant metabolites in the H0 and H24 models, as well as for Δ_H24-H0_, were performed and are shown in Table 5. Accordingly, based on our data, most ROCs for the metabolites showed slightly better performance in the classification of survival than SAPSII and SOFA, not only within the H0 and H24 models, but also with the value of Δ_H24-H0_.

**Table 5:** Area under ROC for key metabolites that separate septic shock survivors from non-survivors

	AUROC_H0_ (*n* = 69)	AUROC_H24_ (*n* = 51)	AUROC_H24-H0_ (*n* = 51)
Lactate	0.74	0.75	0.73
Alanine	0.78	0.78	0.67
Glycoprotein (*N*-acetyl)	0.71	0.60	0.65
Glutamate	0.61	0.81	0.71
Glutamine	0.80	0.70	0.74
Pyruvate	0.81	0.83	0.79
Citrate	0.82	0.72	0.72
Creatinine	0.79	0.69	0.70
Phenylalanine	0.84	0.73	0.79
SOFA	0.60	0.64	0.61
SAPSII	0.62		

*n* number of patients

## Discussion

Effective prognosis can help to improve outcomes for septic shock patients. However, septic shock prognosis can be complicated by patient-specific factors that affect responsiveness to therapy. With the use of metabolomic techniques, we have determined the serum metabolome fingerprint of septic shock patients with both H0 and H24 samples. We also investigate the metabolic footprint along with the evolution from H0 to H24. To our knowledge, our study is the first to reveal time-trend metabolic differences using NMR-based metabolomics between septic shock survivors and non-survivors within 24 h after ICU admission.

### Metabolic variations for H0 and H24 unpaired models separating SSS from SSN

The H0 and H24 unpaired models reveal the differences of metabolome fingerprint between SSS and SSN at the admission to ICU and at 24 h after the admission. Regarding the common discriminatory metabolites found in both models, consistent increases in energy-related metabolites, creatinine, 1-MH, and several amino acids, as well as decreases in glycoproteins are observed as important signals in the non-survivors. Such variations found in SSN at both H0 and H24 are likely to reflect more severe sepsis-induced inflammatory responses and organ dysfunctions that contribute to poor outcomes.

The deregulation of TCA cycle intermediates, such as more concentrated citrate found in the SSN, is one of the consequences of severe stress induced by sepsis [18]. Stress also results in an unregulated catabolism [19]. Enhanced degradation of glycoproteins indicates an aggravated stress in the non-survivors. Also, increases in various amino acids and ketone bodies at H24 in the non-survivors are known as effects of protein breakdown and enhanced lipid oxidation [20]. Notably, ketone bodies have recently been reported to be immune suppressors [21], and elevations in these metabolites may contribute to a negative response in critical illness [22].

Our results also provide evidence for the metabolic variations that are associated with severe organ dysfunction in non-survivors. As shown in Table 1, significantly lower oxygen pressures with higher blood lactate levels indicate the presence of more severe hypoxia in the non-survivors than in survivors. This may be due to mitochondrial disorder, defective TCA cycle [23], which results in dampened aerobic respiration and abnormal energy supply. Severe disorders in energy supply should be an import factor inducing organ failure [24–26]. Other variations involving organ dysfunction are found in creatinine and 1-MH. Their elevations in the comparison in SSN are also supported by some other previous studies [27, 28].

### Different variations for some key discriminators between SSS and SSN H0-H24 multilevel models

As shown in Table 4, different pairwise alterations of relevant metabolites in the comparison between SSS and SSN groups indicate distinct trends in development along with clinical therapy. Interestingly, most of these metabolites related to septic shock evolution are in accordance with the discriminatory molecules found with the H0 and H24 unpaired models. Besides the deregulation of energy-related molecules, increases in four amino acids may be associated with severe protein breakdown and muscle wasting for the non-survivors. Notably, serum concentrations in some amino acids, such as alanine, glutamate, and phenylalanine, are otherwise documented to be involved with hemolysis associated with sepsis [29]. Moreover, as shown in Fig. 4b, glutamate is known to be a core amino acid for conversion into TCA cycle intermediates [30]. Its elevation, as well as the elevation of related amino acids such as glutamine and alanine, is associated with increases in citrate. Phenylalanine can be converted into fumarate. Increases in phenylalanine in patients with poor outcomes have been also reported in other studies [31–33]. The conversion from amino acids to TCA cycle intermediates is likely to provide supplementary energy during severe anoxic conditions, however, is detrimental for the outcomes [34]. Creatinine is known to be an important indicator for monitoring renal injury. Decreases in *N*-acetyl glycoproteins may correspond to a breakdown of proteins. Interestingly, it has been demonstrated by DeCoux et al. [35] that inflammation-induced enriched extracellular glycoproteins are associated with an optimal response during septic shock.

The present study not only provides support for the findings in our previous work, which investigated metabolic differences between SSS and SSN at H0 [8], but also reveals that different evolutions in the first 24 h after admission to ICU between septic shock survivors and non-survivors are linked to variations in metabolites identified by this study. The ROC results shown in Table 5 also show that the key metabolite discriminators are good classifiers for separating SSN from SSS during the first 24 h after ICU admission. We have reason to believe that sustained enrichment of energy-related metabolites and amino acids can provide early warning of a bad outcome.

## Conclusion

In the present study, we have investigated metabolic differences between the survivors and non-survivors of septic shock with the samples obtained at ICU admission and with those obtained 24 h later. We have provided evidence that the sustained enrichment of energy-supply metabolites and amino acids is predictive of a bad outcome. We suggest that monitoring the relevant metabolites in the first 24 h may help to evaluate early therapeutic response.

## Additional files

Additional file 1 **Table S1.** Assignment of spectra recorded with one exemplar serum sample from a septic shock patient. Figure S1. Assignment of spectra recorded with an example of a representative ^1^H-NMR spectrum. The assigned peaks corresponding with the key metabolite discriminants have been marked in the figure. Figure S2. A PCA calculated with H0 samples from 11 nonsurvivors who died during the first 24 h (red dots) and those from the other non-survivors who died from the second day to the seventh day after the first sampling (blue dots). Figure S3. PCA model separating survivors from non-survivors with H0 samples before the exclusion of outlier. One sample of a non-survivor was observed as an outlier for the PCA. This outlier has been removed before statistical analyses. Blue dots: survivors, yellow dots: non-survivors. Figure S4 (respectively S5). Cross-validation by 200 times permutation between *X* and *Y* for the OPLS-DA model with H0 samples (respectively H24). The green dots stand for the obtained *R*^2^ value and the blue dots stand for the obtained *Q*^2^ value within the 200 permutations. The *Y*-axis represents *R*^2^ and *Q*^2^ calculated for every model while the *X*-axis represents the correlation coefficient between original and permuted response data. Figure S6. Loading plots for paired OPLS-DA models showing important discriminatory metabolites that contribute to the separation between H0 and H24 samples. The paired models for the survivors and non-survivors are shown separately. The peaks are assigned to corresponding discriminatory metabolites. The correlations between the assigned metabolites and the model have been shown with the colors. a: loading plot for the separation between H0 and H24 for the survivors; b: loading plot for the separation between H0 and H24 for the non-survivors. (DOCX 829 kb)

## Abbreviations

AUROC: Area under receiver operation curve
FDR: False discovery rate
HB: Hydroxybutyrate
HES: Hydroxyethyl starch
ICU: Intense care unit
JRES: J-resolved spectroscopy
MH: Methylhistidine
MODS: Multi-organ dysfunction syndrome
NMR: Nuclear magnetic resonance
OPLS-DA: Orthogonal projections to latent structures-discriminant analysis
PCA: Principal component analysis
PCT: Procalcitonin
SAPSII: New Simplified Acute Physiology Score
SOFA: Sequential organ failure assessment
SSN: Septic shock non-survivors
SSS: Septic shock survivors
TCA: Tricarboxylic acid
TOCSY: Total correlation spectroscopy
