# Supplementary material for: Nuclear magnetic resonance-based serum metabolomic analysis reveals different disease evolution profiles between septic shock survivors and non-survivors
Source: Crit Care. 2019 May 14;23:169. doi: 10.1186/s13054-019-2456-z (PMC6518644; doi:10.1186/s13054-019-2456-z)
Supplement: Supplementary file 1 — Table S1. Assignment of spectra recorded with one exemplar serum sample from a septic shock patient. Figure S1. Assignment of spectra recorded with an example of a representative 1H-NMR spectrum. The assigned peaks corresponding with the key metabolite discriminants have been marked in the figure. Figure S2. A PCA calculated with H0 samples from 11 nonsurvivors who died during the first 24 h (red dots) and those from the other non-survivors who died from the second day to the seventh day after the first sampling (blue dots). Figure S3. PCA model separating survivors from non-survivors with H0 samples before the exclusion of outlier. One sample of a non-survivor was observed as an outlier for the PCA. This outlier has been removed before statistical analyses. Blue dots: survivors, yellow dots: non-survivors. Figure S4 (respectively S5). Cross-validation by 200 times permutation between X and Y for the OPLS-DA model with H0 samples (respectively H24). The green dots stand for the obtained R2 value and the blue dots stand for the obtained Q2 value within the 200 permutations. The Y-axis represents R2 and Q2 calculated for every model while the X-axis represents the correlation coefficient between original and permuted response data. Figure S6. Loading plots for paired OPLS-DA models showing important discriminatory metabolites that contribute to the separation between H0 and H24 samples. The paired models for the survivors and non-survivors are shown separately. The peaks are assigned to corresponding discriminatory metabolites. The correlations between the assigned metabolites and the model have been shown with the colors. a: loading plot for the separation between H0 and H24 for the survivors; b: loading plot for the separation between H0 and H24 for the non-survivors. (DOCX 829 kb) [file 13054_2019_2456_MOESM1_ESM.docx]

Nuclear magnetic resonance-based serum metabolomic analysis reveals different disease evolution profiles between septic shock survivors and non-survivors

Zhicheng LIU^1,2^, Mohamed N.TRIBA^2^, Roland AMATHIEU^2,3^, Xiangping Lin^2^, Nadia BOUCHEMAL^2^, Edith Hantz^2^, Laurence LE MOYEC^4^, Philippe SAVARIN^2^

1. School of pharmacy, Anhui medical university, Hefei, China
2. Sorbonne Paris Cité, Laboratoire de Chimie, Structures et Propriétés de Biomateriaux et d’Agents Therapeutiques, UMR 7244, University Paris 13 Bobigny, France

3. GH Diaconesse – La Croix Saint Simon – Service de Réanimation, France

4. University of Evry Val d’Essonne, UBIAE, EA7362, Evry, France

Introduction:

Commentaries:

Concerning figure S1: The assignment of spectral signals was performed with Chenomx software in which a database of metabolites was available. All the assigned signals were matched to the metabolites in the database according to the chemical shift (tolerance = 0.02 ppm) and peak shape.

Concerning figure S6:

The directions of peaks are opposite to the directions of

variation of corresponding metabolites during the H0-H24 evolution (The positive signals correspond to the metabolites that are decreased during the H0-H24 evolution; the negative signals correspond to the metabolites that are increased during the H0-H24 evolution).

**Table S1.** Assignment of spectra recorded with one exemplar serum sample from a septic shock patient. s: singlet; d: double; dd: doublet of doublet; t: triplet; q: quartet; m: multiplet.

| 0.85^m^ 1.23^m^ | VLDL, LDL, lipids |
| --- | --- |
| 0.82^d^ 0.95^d^ | 2-Hydroxyisovalerate |
| 0.89^t^ 1.64^m^ 1.73^m^ | 2-HB |
| 0.93^t^ 0.99^d^ | Isoleucine |
| 0.95^dd^ 1.70^m^ | Leucine |
| 0.97^d^ 1.03^d^ | Valine |
| 1.06^d^ | 3-Hydroxyisobutyrate |
| 1.19^d^ 4.15^m^ | 3-HB |
| 1.30^s^ | 3-Hydroxy-3-methylglutarate |
| 1.13^d^ | Isobutryrate |
| 1.16^d^ | Isopropanol |
| 1.22^d^ | Methylmalonate |
| 1.32^d^ 4.11^q^ | Lactate |
| 1.40^m^ | Glycocholate |
| 1.47^d^ | Alanine |
| 1.875^m^ 1.70^m^ | Lysine |
| 1.91^s^ | Acetate |
| 1.98^m^ 3.32^m^ | Proline |
| 1.99^s^ | Acetamide |
| 2.02^s^ | Glycoproteine |
| 2.12^m^ 2.32^m^ | Glutamate |
| 2.15^s^ | Methionine |
| 2.09^m^ 2.41^m^ | Glutamine |
| 2.2^s^ | Acetoacetate |
| 2.36^s^ | Pyruvate |
| 2.39^s^ | Succinate |
| 2.52^d^ 2.68^d^ | Citrate |
| 2.71^s^ | Dimethylamine |
| 2.72^m^ | Lipids (fatty acid residues) |
| 2.89^s^ | Trimethylamine (TMA) |
| 2.90^s^ | N,N-dimethylglycine |
| 3.05t | Proline |
| 3.25^s^ | Creatine |
| 3.03^s^ 3.92^s^ | Creatinine |
| 3.1^s^ | Malonate |
| 3.14^s^ | Dimethysulfone |
| 4.05^t^ | Myo-insitol |
| 5.79^s^ | Urea |
| 6.52^s^ | Fumarate |
| 6.88^d^ 7.18^d^ | Tyrosine |
| 7.32^m^ 7.36^m^ | Phenylalanine |
| 7.77^d^ | Tyrosine |
| 7.67^s^ 7.02^s^ | 1-MH |
| 7.70^s^ | dTTP |

**Supplementary figures:**


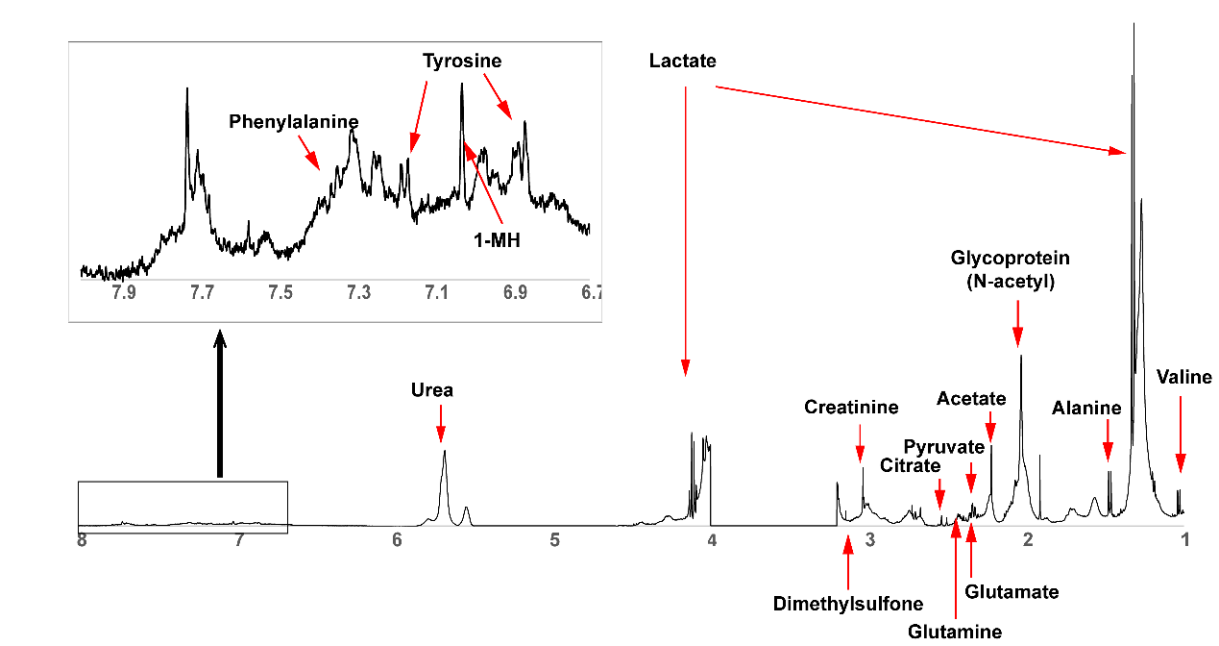


**Figure S1.** Assignment of spectra recorded with an example of a representative ^1^H-NMR spectrum. The assignment of spectral signals was performed with Chenomx Software in which a database of metabolites was available. All the assigned signals were matched to the metabolites in the database according to the chemical shift (tolerance =0.02ppm) and peak shape. The assigned peaks corresponding with the key metabolite discriminants have been marked in the figure.

**
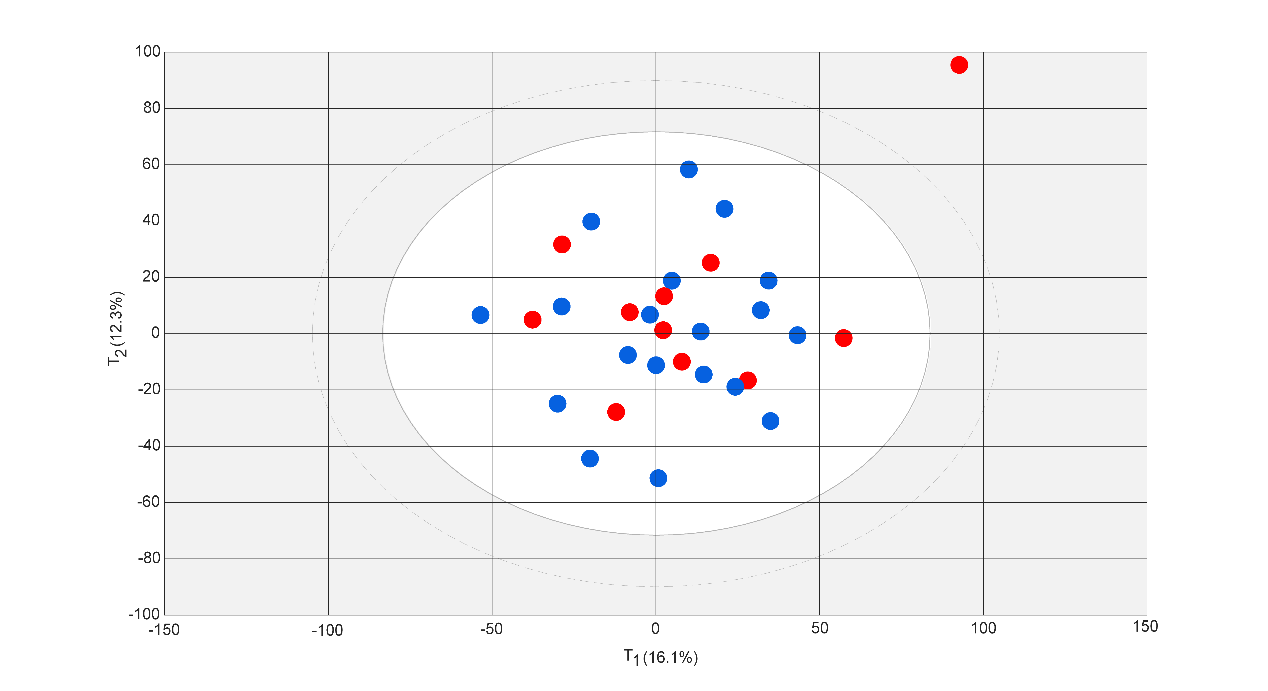
**

**Figure S2.** A PCA calculated with H0 samples from 11 non-survivors who died during the first 24 hours (red dots) and those from the other non-survivors who died from the second day to the 7^th^ day after the first sampling (blue dots).

**
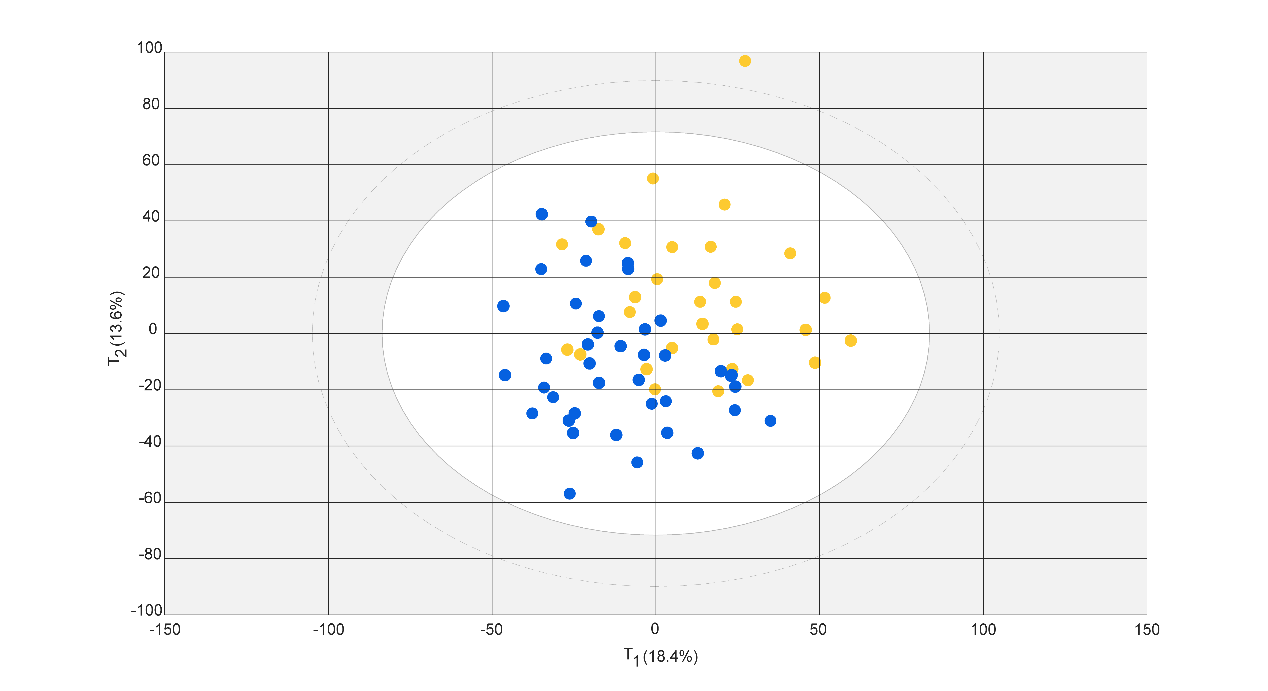
**

**Figure S3** PCA model separating survivors from non-survivors with H0 samples before the exclusion of outlier. One sample of a non-survivor was observed as an outlier for the PCA. This outlier has been removed before statistical analyses. Blue dots: survivors, yellow dots: non-survivors.

**
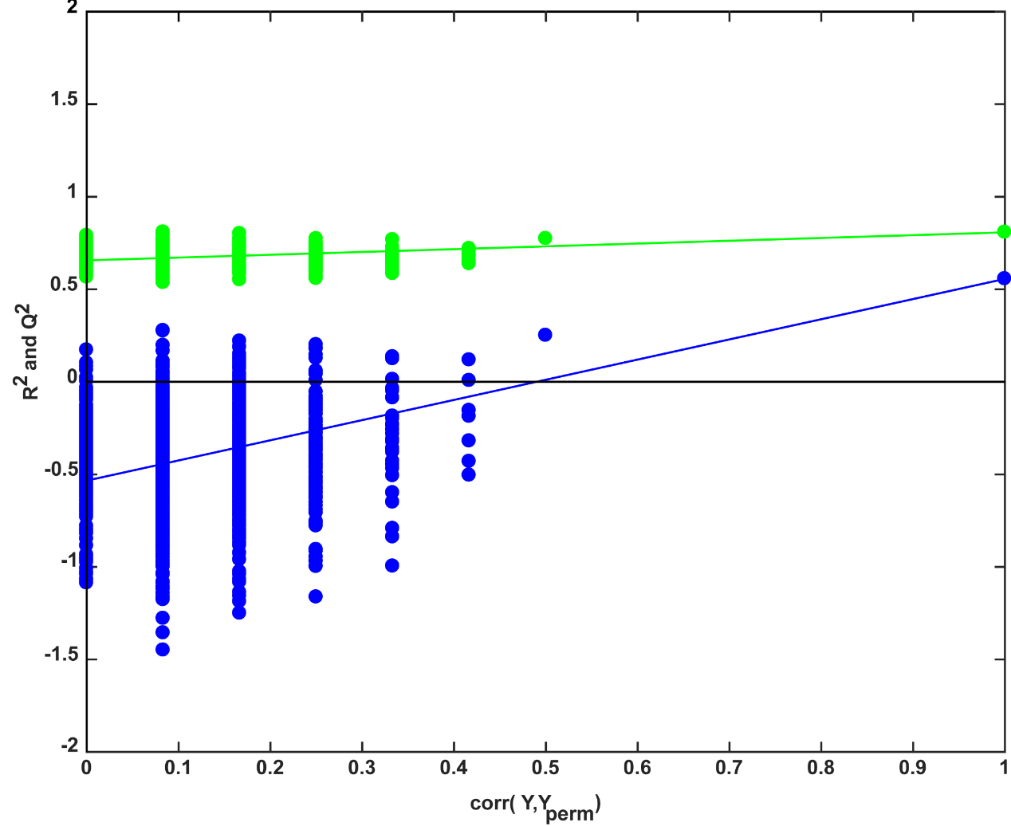
**

**Figure S4.** Cross-validation by 200 times permutation between X and Y for the OPLS-DA model with H0 samples. The green dots stand for the obtained R^2^ value and the blue dots stand for the obtained Q^2^ value within the 200 permutations. The Y-axis represents R^2^ and Q^2^ calculated for every model while the X-axis represents the correlation coefficient between original and permuted response data.

**
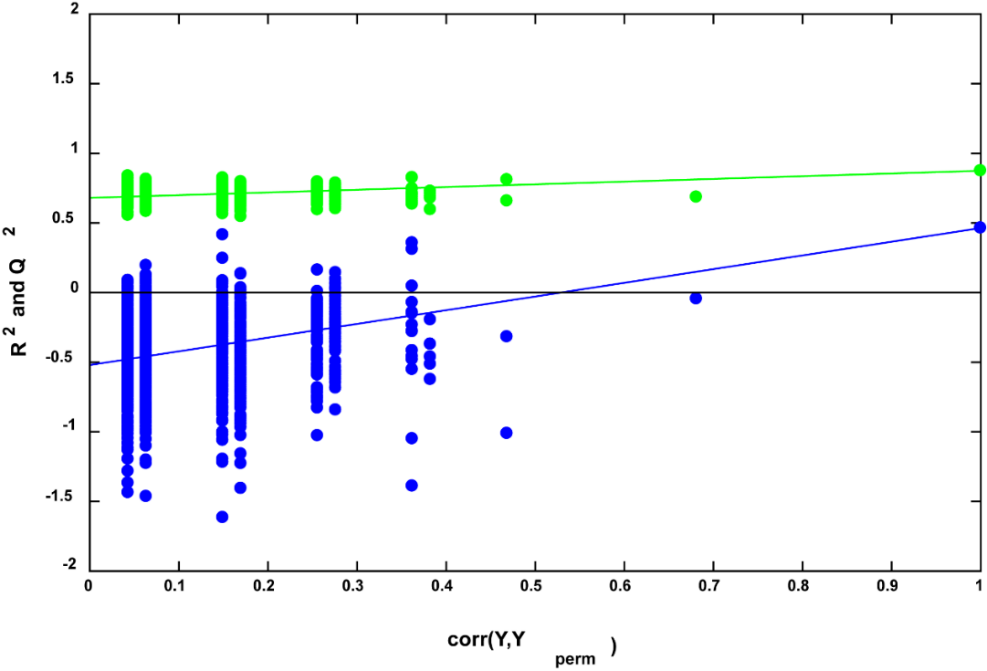
**

**Figure S5.** Cross-validation by 200 times permutation between X and Y for the OPLS-DA model with H24 samples. Cross-validation by 200 times permutation between X and Y for the OPLS-DA model with H0 samples. The green dots stand for the obtained R^2^ value and the blue dots stand for the obtained Q^2^ value within the 200 permutations. The Y-axis represents R^2^ and Q^2^ calculated for every model with permutation while the X-axis represents the correlation coefficient between original and permuted response data.

**
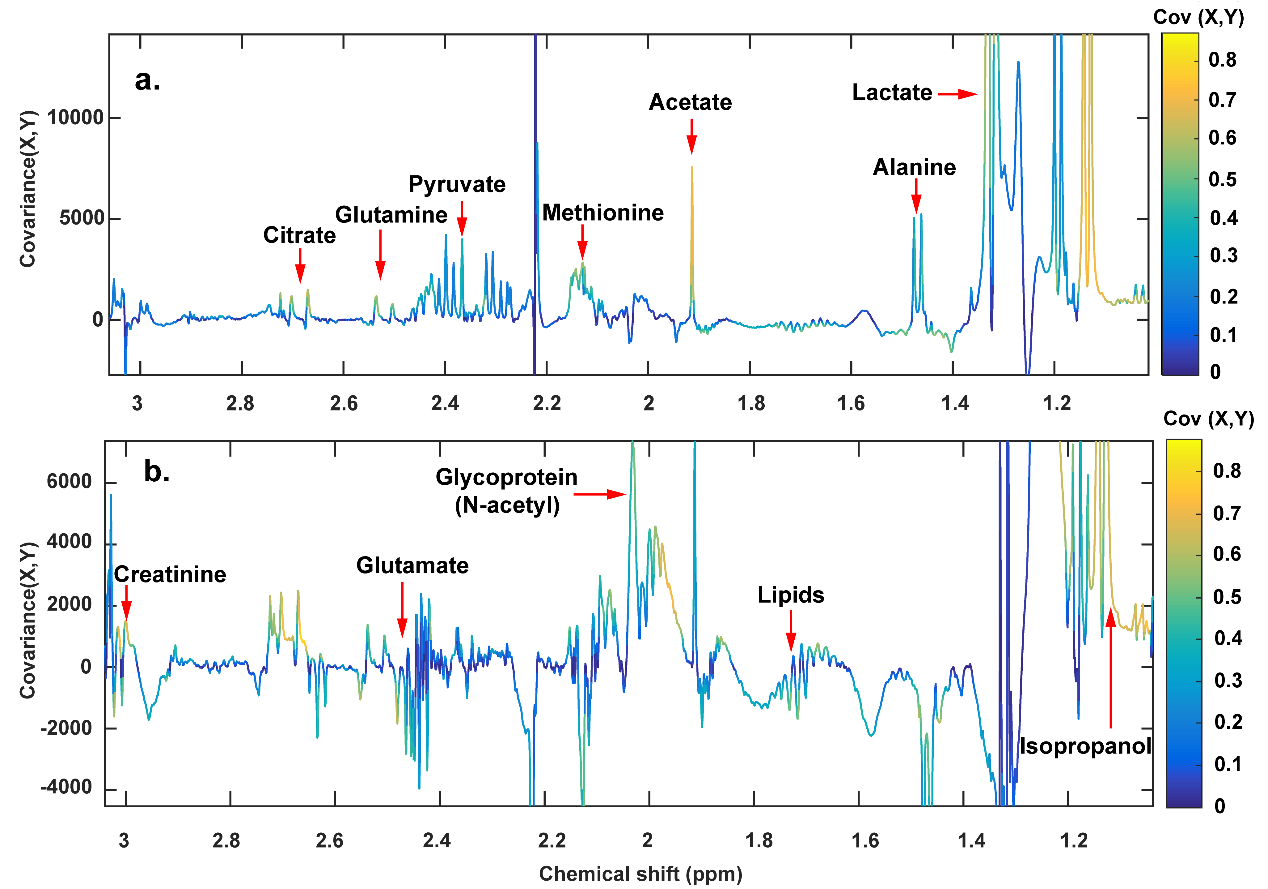
**

**Figure S6.** Loading plots for paired OPLS-DA models showing important discriminatory metabolites that contribute to the separation between H0 and H24 samples. The paired models for the survivors and non-survivors are shown separately. The peaks are assigned to corresponding discriminatory metabolites. The correlations between the assigned metabolites and the model have been shown with the colors. a: loading plot for the separation between H0 and H24 for the survivors; b: loading plot for the separation between H0 and H24 for the non-survivors. The directions of peaks are opposite to the directions of variation of corresponding metabolites during the H0-H24 evolution (The positive signals correspond to the metabolites that are decreased during the H0-H24 evolution; the negative signals correspond to the metabolites that are increased during the H0-H24 evolution).
